# The California Cognitive Assessment Battery (CCAB)

**DOI:** 10.3389/fnhum.2023.1305529

**Published:** 2024-01-11

**Authors:** David Woods, Peter Pebler, David K. Johnson, Timothy Herron, Kat Hall, Mike Blank, Kristi Geraci, Garrett Williams, Jas Chok, Sandy Lwi, Brian Curran, Krista Schendel, Maria Spinelli, Juliana Baldo

**Affiliations:** ^1^NeuroBehavioral Systems Inc., Berkeley, CA, United States; ^2^Department of Neurology, University of California, Davis, Davis, CA, United States; ^3^VA Northern California Health Care System, Martinez, CA, United States

**Keywords:** memory, attention, aging, executive function, automatic speech recognition, remote assessment, processing speed, dementia

## Abstract

**Introduction:**

We are developing the California Cognitive Assessment Battery (CCAB) to provide neuropsychological assessments to patients who lack test access due to cost, capacity, mobility, and transportation barriers.

**Methods:**

The CCAB consists of 15 non-verbal and 17 verbal subtests normed for telemedical assessment. The CCAB runs on calibrated tablet computers over cellular or Wi-Fi connections either in a laboratory or in participants’ homes. Spoken instructions and verbal stimuli are delivered through headphones using naturalistic text-to-speech voices. Verbal responses are scored in real time and recorded and transcribed offline using consensus automatic speech recognition which combines the transcripts from seven commercial ASR engines to produce timestamped transcripts more accurate than those of any single ASR engine. The CCAB is designed for supervised self-administration using a web-browser application, the Examiner. The Examiner permits examiners to record observations, view subtest performance in real time, initiate video chats, and correct potential error conditions (e.g., training and performance failures, etc.,) for multiple participants concurrently.

**Results:**

Here we describe (1) CCAB usability with older (ages 50 to 89) participants; (2) CCAB psychometric properties based on normative data from 415 older participants; (3) Comparisons of the results of at-home vs. in-lab CCAB testing; (4) We also present preliminary analyses of the effects of COVID-19 infection on performance. Mean z-scores averaged over CCAB subtests showed impaired performance of COVID+ compared to COVID- participants after factoring out the contributions of Age, Education, and Gender (AEG). However, inter-cohort differences were no longer significant when performance was analyzed with a comprehensive model that factored out the influences of additional pre-existing demographic factors that distinguished COVID+ and COVID- cohorts (e.g., vocabulary, depression, race, etc.,). In contrast, unlike AEG scores, comprehensive scores correlated significantly with the severity of COVID infection. (5) Finally, we found that scoring models influenced the classification of individual participants with Mild Cognitive Impairment (MCI, z-scores < –1.50) where the comprehensive model accounted for more than twice as much variance as the AEG model and reduced racial bias in MCI classification.

**Discussion:**

The CCAB holds the promise of providing scalable laboratory-quality neurodiagnostic assessments to underserved urban, exurban, and rural populations.

## Introduction

Traditional, manually administered neuropsychological assessments (NPAs) have several shortcomings: They are costly, time-consuming to administer and score, and suffer from examiner effects and scoring errors that limit their precision and reliability. Moreover, access to manual NPAs is limited by a shortage of licensed clinicians to administer, score, and interpret test results. Computerized NPAs can address these shortcomings (Wild et al., 2008; Cole et al., 2018; Rao, 2018; Bilder and Reise, 2019; Sternin et al., 2019; Tsoy et al., 2021; Ashford et al., 2022; Jutten et al., 2022; Libon et al., 2022; Tavabi et al., 2022; Wilmoth et al., 2022). However, with rare exceptions (Mackin et al., 2022), existing computerized NPAs do not incorporate verbal tests which constitute the majority of manually administered cognitive tests (Rabin et al., 2016).

The lack of verbal tests limits the clinical use of existing digital NPAs because verbal memory tests, with spoken verbal stimuli and spoken responses, are routinely used to detect the hallmark symptoms of mild cognitive impairment (MCI), Alzheimer’s Disease and Related Disorders (ADRD), and preclinical dementia (Weintraub et al., 2018; Vyhnalek et al., 2022). Thus, the lack of verbal tests impedes the adoption of computerized NPAs. In addition, most remote computerized NPAs are self-administered and do not permit the examiner to observe the patient or monitor performance during testing. Patient monitoring is essential for assuring test validity, particularly when evaluating more severely disabled patients who may fail to understand test instructions or unexpectedly stop performing.

**FIGURE 1:**
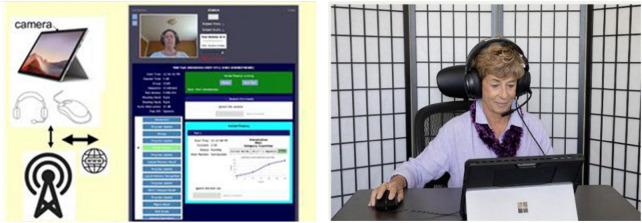
The CCAB is administered on a standard PC tablet computer with cellular connectivity (**left**) so that participants can be tested at home (**right**) or in the laboratory. The examiner can initiate video chats with participants and monitor their performance in real time (**middle**).

Here, we describe the California Cognitive Assessment Battery (CCAB) which addresses these limitations. The CCAB is a novel battery of empirically validated subtests designed for remote assessment, either at-home or in the laboratory (see Figure 1). Unlike most digital NPAs, the CCAB enables examiners to remotely control test delivery, monitor test performance in realtime, observe and talk to the patient, and correct test-administration problems. The CCAB standardizes test administration, reduces testing time and patient burden, lessens examiner bias and error, and accurately transcribes speech to tally and score both verbal responses and verbal response latencies. First, we describe the history of CCAB development, the features of individual CCAB subtests, and their psychometric properties. Then we describe CCAB scoring and analysis procedures and compare the results of at-home and in-lab testing. Lastly, we demonstrate CCAB use in the preliminary analysis of the effects of COVID-19 on cognition and show how different scoring models impact the MCI classification of patients with impaired performance.

### Development of the California cognitive assessment battery (CCAB)

Prototype CCAB subtests (finger tapping, simple reaction time, and digit span) were developed in 2010 to improve the objectivity and precision of cognitive testing in a large scale epidemiological study of the effects of hydrogen sulfide (Reed et al., 2014). The CCAB subtests were programmed with Presentation^®^ software developed by Neurobehavioral Systems (NBS, Berkeley, CA, USA). Several Presentation^®^ features were important in these early efforts. (1) Presentation^®^ uses a 100 kHz clock to record stimulus and response events with calibrated sub-millisecond precision. This feature helped resolve a controversy over purported declines in processing speed caused by dysgenics and environmental pollution (Woods et al., 2015f). (2) Existing computerized test batteries do not detect timing errors during test execution due to the competition for CPU resources from other processes or viruses. Presentation^®^ measures the timing precision of each test event to detect abnormal hardware and software conditions that increase temporal imprecision and can invalidate test results (Plant, 2016). (3) Presentation^®^ algorithms are robust and extensively tested. Each new version of Presentation^®^ undergoes more than 24,000 quality assurance tests on Windows, iOS, and Android operating systems. Moreover, Presentation^®^ is used in thousands of research laboratories around the world so that subtle bugs that escape QA discovery can be detected, corrected, and prevented. Thus, Presentation^®^ provides a robust platform for cognitive test development.

### Automating CCAB testing

In 2018, NBS received a fast-track SBIR grant (NIA, R44AG062076) to automate and expand the original CCAB battery which had previously required examiners to explain test procedures verbally and manually score verbal responses. We made four major changes to enable fully automated test delivery and scoring:

1. Natural sounding “neural” text-to-speech voices (Microsoft, Redmond, WA, USA) were incorporated into Presentation^®^ to deliver subtest instructions and verbal stimuli.
2. Practice trials were automated to assure that participants understood subtest instructions and performed at criterion levels before testing began.
3. ASR capabilities were added to Presentation^®^ to enable preliminary verbal subtest scoring in real time.
4. The Examiner interface was developed to allow for remote test administration, control, and monitoring.

### CCAB hardware

The CCAB is currently administered on a Microsoft Surface Pro tablet with a touchscreen, mouse, and packaged in a compact carrying case for delivery to participants’ homes. All tablets have built-in cellular and Wi-Fi connectivity. Auditory stimuli are delivered through noise-attenuating circumaural headphones (V20U, Jeecoo, Shenzhen, PRC) that include a noise-canceling head-mounted microphone for recording speech. A computer gaming mouse (Taipan, Razer, Irvine, CA, USA) is used on subtests requiring button-press responses (Figure 1). CCAB stimulus delivery and scoring is controlled by the local test tablet, so that test administration is unaffected if cellular or Wi-Fi connectivity is interrupted. To make home testing feasible regardless of examinees’ digital literacy, CCAB test kits include easy to follow setup instructions. Start-up requires a single tap on the tablet home screen after powering on, with instructions and tests automatically presented thereafter.

### The Examiner interface

Neurobehavioral Systems developed the Examiner interface to enable examiners to monitor participants during testing and intervene if stress, fatigue, or technical problems were detected (Pebler et al., 2022). The Examiner interface runs in a web browser and enables examiners to monitor test administration and initiate video or audio chats (Figure 1). The Examiner interface also communicates with the test computer to report tablet error conditions (e.g., low tablet power), and participant performance problems (e.g., training failures, failures to successfully begin a subtest, etc.). The Examiner displays subtest performance in real time (Figure 1, center), records timestamped examiner observations, and enables the examiner to pause, halt, and restart subtests. Finally, multiple examiners can simultaneously view an active test session, a feature that is useful for examiner training and in helping resolve problems that are challenging for inexperienced examiners.

Cellular connectivity is built into each test station and is essential for reaching underserved populations. According to the Community Tech Network, nearly 22 million (42%) older Americans lack at-home broadband access (Singh et al., 2020; Lin et al., 2022). Moreover, Black and Latino seniors are, respectively, 2.5 and 3.3 times more likely to lack this broadband access compared to whites. Thus, medical-grade remote NPAs require a test platform with built-in cellular as well as Wi-Fi connectivity.

### Automated verbal subtest scoring

**FIGURE 2:**
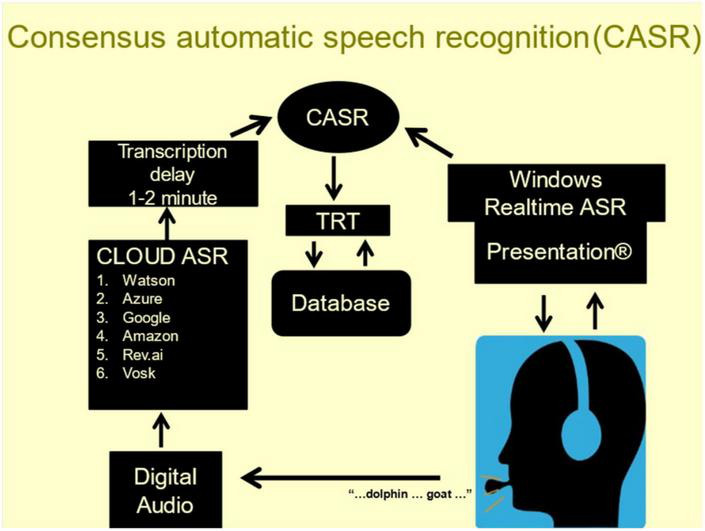
Examinees wear a calibrated head-mounted microphone. Speech (**bottom**) is transcribed by realtime ASR (top **right**) and digitally recorded (bottom **left**). The recordings are sent to six cloud-based ASR engines (middle **left**). The individual transcripts are temporally aligned and combined using task-specific weighted voting to select the most likely word along with a confidence metric reflecting the probability of transcription error.

To improve the accuracy of automated verbal subtest scoring, NBS developed consensus ASR (CASR, Figure 2), a cloud-based speech-transcription pipeline (Woods et al., 2022). CASR utilizes ROVER methodology (Fiscus, 1997) to integrate the information from six commercial ASR engines and the realtime engine by using weighted voting to select the optimal consensus transcription after aligning words using the Levenshtein algorithm (Levenshtein, 1966).

Consensus automatic speech recognition transcription accuracy exceeds that of any single ASR engine for subtests with simple verbal responses (∼99% accuracy) and for subtests eliciting discursive speech (∼95% accuracy) (Woods et al., 2022). Moreover, CASR also provides a confidence metric for each transcribed word based on the concordance of the transcriptions from the individual ASR engines. Finally, CASR similarly combines the word timestamps from individual ASR engines to produce consensus timestamps that quantify verbal response latencies and provide speech-timing measures (hesitations, temporal word clustering, etc.) used to supplement the keyword scoring in subtests that generate discursive speech (e.g., logical memory).

### Transcript review and correction

**FIGURE 3:**
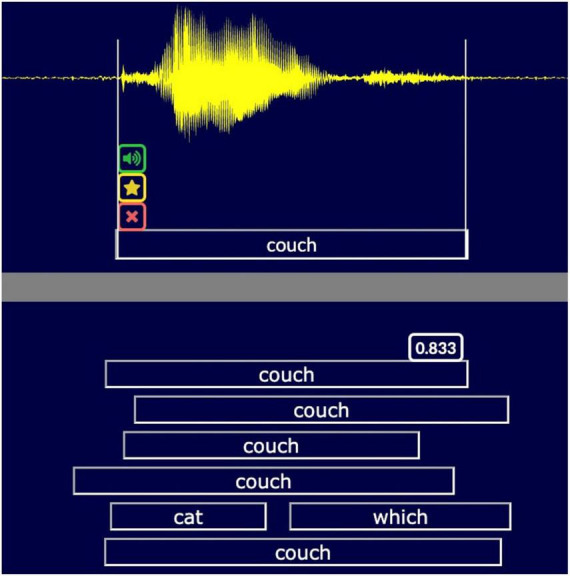
The transcript review tool (TRT). A snippet of the TRT screen showing the transcription of the word “couch.” The TRT displays the waveform (**top**), the corrected CASR transcription (**below** waveform) and the different transcripts (uncorrected CASR, with confidence metric, RevAI, MS Azure, Google, Watson, and Vosk). Examiners can replay and edit words, flag, or delete words, and adjust timestamps (vertical white lines). A spectral view is optional.

To evaluate CASR accuracy we developed a transcript review tool (TRT, Figure 3). The TRT displays the aligned outputs of the individual ASR engines, the consensus transcription of CASR, and the waveform of the participant’s speech. It also provides playback controls for replaying segments of the recording and editing tools for correcting transcript errors. The TRT can advance directly to words transcribed with low CASR confidence, speeding review by skipping words unlikely to have errors. Importantly, TRT files provide a record of the transcription errors to further refine CASR algorithms.

### Current CCAB subtests

From 2018 to 2021, NBS revised nine prototype CCAB subtests and programmed 23 new subtests to assess a broader range of cognitive functions. Most CCAB subtests are brief: the median subtest duration (including introductions and training trials) is 3.2 min, with 77% of subtests lasting less than 5 min. Subtests are separated by 20 s pauses, with 4-min rest periods occurring at 30-min intervals.

During normative data collection, the CCAB was administered in three test sessions on separate days, each requiring ∼90 min. Since 2021, more than 1,000 older participants (ages 50–89) have been tested, most undergoing 3 days of normative data collection. One set of subtests and questionnaires are presented on the first day of testing (Table 1) and a second set of subtests are presented on days 2 and 3 (Table 2) and repeated at 6- and 18-month retests thereafter. As described below, the CCAB includes questionnaires that gather extensive demographic information and verbal and non-verbal subtests of memory, processing speed, and executive function.

**TABLE 1:** Day 1 test–retest reliabilities, showing Pearson correlations across repeated test sessions.

Test	Accuracy	Speed
Auditory threshold	0.69	0.46
ASR quality assurance	0.54	0.91
**Questionnaires (14 min)**		
BAVLT (8 words) DR	0.73	0.59
Logical memory DR	0.85	0.71
VF- 4 categories	0.91	0.57
Figure copy	0.53	0.77
Picture description	0.78	0.96
Picture description DR	0.79	0.93
Vocabulary	0.84	0.68
Stroop compatible	0.89	0.82
Stroop incompatible	0.86	0.83
Finger tapping		0.87
Simple reaction time		0.82
Choice reaction time	0.73	0.77
CPN	0.74	0.87

BAVLT, bay area verbal learning test (8-word version); VF, verbal fluency; DR, delayed recall; CPN, continuous picture naming. Subtest order was different than shown. Data are from 100 participants who underwent repeated Day 1 testing.

**TABLE 2:** Day 2/3 test–retest reliabilities.

Test	Accuracy	Speed
BAVLT	0.84 (0.83)	0.56 (0.58)
FNAME binding	0.83 (0.80)	0.54 (0.63)
Logical memory	0.81 (0.71)	0.62 (0.69)
Semantic Stroop	0.79 (0.76)	0.81 (0.83)
Picture naming	0.78 (0.67)	0.80 (0.77)
VF (animals)	0.77 (0.68)	0.56 (0.59)
VF (vegetables)	0.74 (0.68)	0.43 (0.43)
DS forward	0.64 (0.66)	0.69 (0.60)
DS reverse	0.69 (0.64)	0.73 (0.74)
Symbol number	0.38 (0.43)	0.77 (0.79)
Figure copy	0.63 (0.64)	0.75 (0.64)
Design fluency	0.7 5 (0.74)	0.76 (0.75)
Spatial span	0.48 (0.41)	0.77 (0.68)
Hidden patterns	0.81 (0.77)	0.76 (0.67)
Identical pictures	0.90 (0.86)	0.88 (0.84)
Trails A	0.28 (0.24)	0.76 (0.65)
Trails B	0.56 (0.26)	0.76 (0.65)
Mental rotation	0.67 (0.61)	0.82 (0.75)
CPN	0.81 (0.66)	0.70 (0.74

DS, digit span; FNAME, face-name binding. Pearson correlations between Day 2 and Day 3 test sessions are shown with correlations between Day 3 and 6-month retest shown in parentheses. Subtest order was different than shown. Data from 415 participants. See Table 1 for more abbreviations.

**FIGURE 4:**
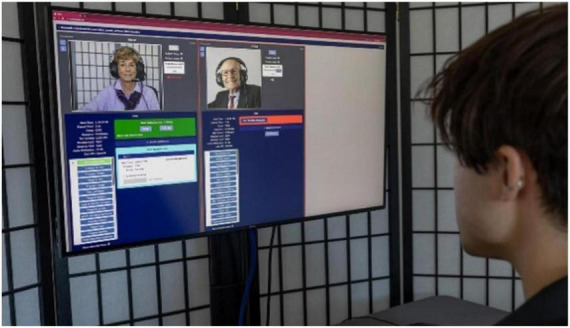
An examiner monitoring two concurrent CCAB tests sessions.

The CCAB’s standardized test instructions and practice trials have enabled the successful testing of more than 99% of all participants, with <1% of CCAB subtests requiring examiner intervention (Chok et al., 2022). CCAB automation and error reporting also enhance efficiency by permitting a single examiner to concurrently monitor CCAB test administration to multiple (up to 6) participants (Figure 4). Importantly, the Examiner interface allows examiners to administer tests nationally and internationally from any browser-capable device (including cell phones).

## CCAB subtests

California Cognitive Assessment Battery subtests can be divided into four categories: (1) Subtests to gather demographic and descriptive data to document participant characteristics including questionnaires, measures of sensory thresholds, measures of speech clarity, and a Vocabulary test; (2) Processing speed subtests that measure the response speed of mouse, touch, drawing, and verbal responses in visual and auditory modalities; (3) Memory subtests that measure episodic, associative, and working memory in visual and auditory modalities; and (4) Executive function subtests in verbal and visuospatial domains.

### Demographic and descriptive data

#### Questionnaires

California Cognitive Assessment Battery questionnaires administered at enrollment require approximately 14 min to complete and gather extensive demographic information including age, gender, education, handedness, geographical region of upbringing, race, ethnicity, linguistic background, health conditions, marital status, drug and alcohol use, family history of dementia and mental illness, socioeconomic and marital status, COVID infections (including dates, severity, and long COVID symptoms), sleep, alertness, overall health and wellbeing, and weekly exercise. Additional inventories include the Geriatric Depression Scale (GDS-15) (Sheikh and Yesavage, 1986), General Anxiety Disorder (GAD-7) scale (Spitzer et al., 2006), Cognitive Failures Questionnaire (CFQ), a subjective measure of cognitive problems (Broadbent et al., 1982), and FS20 measures of functional disability (Jette et al., 1986). Question completion time (QCT), a measure of executive function (Woods et al., 2015h), is recorded for each question.

#### Sensory thresholds

Auditory and visual threshold assessments are used to assure that performance reflects cognitive ability rather than hearing or visual loss. Word recognition thresholds assess speech recognition thresholds. Auditory stimulus intensities (normally 75 dB SPL) are then adjusted to correct for hearing loss (Füllgrabe, 2020) during the remaining tests. Visual acuity thresholds quantify the minimal legible text font to assure that participants’ vision is sufficient for CCAB testing.

#### Speech transcription accuracy

Consensus automatic speech recognition transcription accuracy and reading rate are measured as participants read aloud a prose passage and lists of numbers and words to assure valid verbal response scoring.

#### Vocabulary

A multiple-choice vocabulary test adaptively samples tokens from a 600-word corpus ordered in difficulty by over 40,000 visitors on a popular internet website^1^ and subsequently modified by NBS staff for US populations (Woods et al., 2022). RTs are measured and vocabulary is quantified with psychophysical procedures (Killion et al., 2004). Preliminary studies and unpublished simulations show that the CCAB ’s 4-min, 24-word adaptive vocabulary subtest provides more accurate and replicable estimates of vocabulary than the WAIS 30-min manually administered vocabulary test. Vocabulary tests reflect estimated premorbid cognitive capacity and hence serves as an important reference for evaluating baseline cognition and cognitive reserve (Soldan et al., 2017; Summers et al., 2017).

### Processing speed

#### Finger tapping

Participants click the mouse button using their index finger as fast as possible over 20 s. One trial is performed with each hand. Standard metrics include tap count and inter-tap timing with additional measures as described in previous studies (Hubel et al., 2013a,b).

#### Simple reaction time (SRT)

A bullseye target stimulus is briefly presented to either the left or right hemifield at varying interstimulus intervals. Participants respond as quickly as possible to each target using the mouse. Standard metrics are RT and accuracy, with additional measures as described in previous studies (Woods et al., 2015e,f).

#### Choice reaction time (CRT)

A letter (P or F) in one of two possible colors (blue, orange) is presented in either the left or right hemifield. Participants respond with the mouse, clicking the one button for the target (blue P) and the other for the three distractors. The stimulus onset asynchrony (SOA) is adaptive, decreasing after hits and increasing after incorrect responses. Standard metrics include RT and the minimum SOA reached, with additional measures as described in previous studies (Woods et al., 2015e,g).

#### Stroop

Displays of 36 words are presented in each of four conditions: (1) Color naming of neutral word colors; (2) Color naming with word-compatible colors (e.g., “red” appears in red font); (3) Color naming with word-incompatible colors (e.g., “red” appears in green font); and (4) Word-reading of color names written in black-and-white. Core measures quantify accuracy and RTs. Comparisons of incompatible and compatible conditions are also used to assess executive function.

#### Continuous picture naming (CPN)

Participants see a grid of 24 colored pictures of objects and name each object as quickly as possible. Picture locations are then shuffled, and the process is repeated. Accuracy and response times are measured. Different pictures are used on Day 1 and Days 2/3 of testing.

#### Trails A

Twenty-five numbered circles are presented on the screen, and participants use their finger to draw lines connecting the circles in sequential order. Standard metrics include completion time and error count, with additional measures as described in previous studies (Woods et al., 2015c).

#### Identical pictures

Adapted from Ekstrom et al. (1976), participants are shown an exemplar image (e.g., a smiley face line drawing) and four similar images and asked to select the exemplar as rapidly as possible with a touch response. Six trials are presented on each screen, with new screens displayed automatically. The test continues for 2 min. Scoring includes the number of correct and incorrect trials and mean RTs.

#### Hidden patterns

Adapted from Ekstrom et al. (1976). Participants are shown an exemplar geometric figure and asked to identify test images that contain the exemplar. There are 32 test images per screen. New screens display automatically, and the test continues for 2 min. Scoring includes the number of correct responses and mean RTs.

#### Mental rotation

Participants decide if a brief visual stimulus is the letter R or a mirror-reversed “Я.” Fifty stimuli are presented at five rotation angles. Participants use the mouse to respond. Accuracy and RTs are measured (Schendel et al., 2022).

#### Symbol-number coding

Participants are presented with an array of 12 number/symbol pairs at the top of the screen. Below, they are shown an array of symbols and asked to articulate the matching number for each symbol as quickly as possible. Three 12-symbol trials with different symbol orders are presented. Accuracy and response times are measured.

### Memory

#### Bay area verbal learning test (BAVLT)

There are two versions of the BAVLT (Williams et al., 2022). On day 1, an 8-word list is repeated on two trials, with immediate recall after each list presentation and delayed recall 30 min later. On days 2 and 3, a 12-word list is presented on three trials with immediate recall after each list presentation. This is followed by a distractor list of 12 words, and then the uncued recall of the original list. Delayed recall and recognition are tested 30 min later. Standard metrics include recall totals and word articulation rates, with additional measures described in previous studies (Woods et al., 2017).

#### Picture description

Participants describe a colored picture of a family created for the CCAB (“the slipper thief”–a dog stealing a slipper as family members engage in other activities). After 20 min, participants are asked to recall the scene from memory. Key words and multiple linguistic measures (e.g., speech rate, type/token ratios, word frequencies, etc.) are scored.

#### Face-name binding

This subtest is an abbreviated version of the Face Name associative binding test (Rentz et al., 2011). Participants are shown six faces on at a time, each paired with spoken first and last names and occupations. They recall the first name, last name, and occupation of each individual on two encoding and two immediate recall trials. Delayed recall and recognition are tested 30 min later. Accuracy and response latencies are quantified.

#### Logical memory

On day 1, the participant reads a 109-word story aloud and immediately recalls the story. Delayed recall occurs 30 min later. On days 2 and 3, the participant listens to a different 107-word story and immediately recalls the story. Delayed recall and recognition trials occur 30 min later. Core measures quantify keyword recall and speaking rate.

#### Forward digit span

An auditory digit sequence is presented, after which participants repeat the sequence aloud in identical order. List length is adapted using a 1:1 staircase. Standard metrics include mean span and digit production rate, with additional measures as described in previous studies (Woods et al., 2011a,b).

#### Reverse digit span

An auditory digit sequence is presented, after which participants repeat the sequence aloud in reverse order. List length is adapted using a 1:1 staircase. Standard metrics include mean span and digit production rate with additional measures as described in previous studies (Woods et al., 2011a,b).

#### Spatial span

In Spatial Span, an array of 10 red boxes is presented on screen. An animated cursor highlights a sequence of boxes, after which participants touch boxes in the same order. Span length is adapted using a 1:2 staircase. Standard metrics include mean span and touch latencies, with additional measures as described in previous studies (Woods et al., 2015b,d).

#### Figure copy

Participants copy an abstract black and white figure with their finger on the tablet sceen and reproduce it from memory 30 min later. Core measures include drawing time, segment order, and computationally scored drawing accuracy. Different versions are administered on day 1 and days 2/3.

### Executive function

#### Verbal fluency

On day 1, participants produce as many words as possible in 60 s for each of four different semantic categories (Baldo et al., 2022). On days 2 and 3, two different semantic categories are used. Standard metrics include the number of unique in-category responses and inter-response intervals, with additional measures as described in previous studies (Woods et al., 2016a).

#### Trails B

Twenty-five circles are presented on the screen with letters or numbers. Participants use their finger to draw lines connecting the circles in alternating sequential order (i.e., 1, A, 2, B, etc.). Standard metrics include completion time and error count, with additional measures as described in previous studies (Woods et al., 2015c).

#### Design fluency

In Design Fluency, five circles are presented on screen. Participants use their finger to draw lines connecting the circles to form a unique pattern of four lines, after which the lines are cleared and a new pattern can be drawn. The test continues for 60 s. Standard metrics include the number of unique legal patterns drawn and pattern completion time, with additional measures as described in previous studies (Woods et al., 2016b).

#### Semantic Stroop

Single words are presented in blue or red font. Examinees are asked to produce a synonym for words in blue font and an antonym for words in red font. After 16 trials, the same 16 words are presented again, but in the opposite colors. Error rates and RTs are analyzed.

## Results

### Test–retest reliabilities (TRRs)

Tables 1, 2 show TRRs for the principal accuracy and speed measures from the CCAB subtests. Table 2 also shows TRRs between day 3 and the subsequent 6-month retest in parentheses. TRRs were high for the core measures of most CCAB subtests with occasional lower reliabilities due primarily to ceiling or floor effects that truncated the range of performance (e.g., few errors in symbol-number coding). Overall, TRRs for core measures (accuracy or speed depending on the focus of the subtest) averaged *r* = 0.83 for day 1 subtests and *r* = 0.77 for day 2 and 3 subtests. TRRs between day 3 and 6-month retest were also high (shown in parentheses in Table 2). The high TRRs reflect in part the fact that identical stimuli are presented during the repeated administration of most subtests. Predictable differences in TRRs were also evident for different versions of similar subtests. For example, the TRR of BAVLT delayed recall was lower for the 8-word list on day 1 (*r* = 0.73) than for the 12-word list on days 2/3 (*r* = 0.84), due in part to ceiling effects on the 8-word list. Similarly, the TRRs of mean performance scores averaged over four verbal fluency categories on day 1 was higher (*r* = 0.91) than the single-category scores on days 2 and 3 (*r* = 0.77 and *r* = 0.74). Note that the TRRs on verbal subtests were obtained from unreviewed CASR transcripts; TRRs are expected to increase slightly when ongoing manual transcript review has been completed.

### Feasibility of CCAB testing in healthy older adults

Normative CCAB longitudinal data were collected on 415 older individuals (ages 50–89), including 185 older Veterans, whose demographic characteristics are shown in Table 3 (below). The participants were tested for three days at baseline, including repeated administration of the same subtests on days 2 and 3 to evaluate test-retest reliability and retest learning effects. Follow-up longitudinal assessments with day 2 subtests were performed at 6- and 18-months post-baseline. At-home testing with the CCAB proved feasible: 99.7% of participants successfully completed three days of enrollment testing, with fewer than 3% of participants experiencing setup problems that required extensive examiner intervention. Test failure rates for individual subtests were low: <4% on the most difficult subtest and <1% overall. Participant satisfaction with the CCAB was high due to the comfort and convenience of testing at home and the ability to flexibly schedule test sessions (Chok et al., 2022). Retention was also high with a 10.5% attrition rate at 6-month follow-up, due primarily to medical issues.

**TABLE 3:** Demographic characteristics of subjects.

	*n*	Age	Male%	NW	GAD7	GDS	EDU	fs20	OMNI
COVID−	352	70.47	53%	35%	1.93	1.77	16.07	4.50	0.05
COVID+	63	68.05	35%	68%	3.43	2.59	13.22	5.24	−0.29

The 63 COVID + participants were younger, more often female, more often from racial minorities (NW = non-white), had higher anxiety (GAD7), depression (GDS), and functional status (FS20) scores, and had less education (EDU) than COVID- participants (all *p* < 0.05). Their unadjusted performance scores, averaged over all subtests (OMNI), were also significantly below those of COVID- participants (*p* < 0.02).

### Co-norming of CCAB and manual tests

As part of a CCAB sub-study in healthy Veterans, we are comparing CCAB scores and scores on traditional in-person neuropsychological testing in a population of 90 healthy older Veterans. Data collection and analysis are currently ongoing, but preliminary analyses from 58 Veterans show strong correlations, suggestive of good construct validity: e.g., CCAB Face-Name delayed recall vs. delayed recall vs. the much longer Face Name Associate Memory Exam, *r* = 0.64, *p* < 0.001; Logical Memory delayed recall vs. WAIS Logical Memory delayed recall *r* = 0.55, *p* < 0.001; CCAB Trails B vs. traditional Trail Making B, *r* = 0.71, *p* < 0.001; and CCAB Vocabulary vs. WAIS Vocabulary, *r* = 0.75, *p* < 0.001.

### Summary score calculations

Preliminary analyses show that CCAB test performance was affected by age, education, and gender (AEG): Younger age, higher education, and female gender were associated with better performance. However, additional demographic factors correlated significantly with performance on many CCAB subtests, including digital literacy (i.e., daily computer use), GDS scores, race, weekly hours spent reading, quadratic age effects (age^2^), daily prescription medications, and, most significantly, scores on the CCAB Vocabulary subtest, a 4-mi adaptive test that reflects crystallized knowledge and that improves significantly with age.

In the preliminary analyses reported below, 70 selected subtest scores were scaled and averaged with R (Version 4.1.1, “Kick Things”) to obtain a measure of mean performance (the omnibus score) as well as unregressed mean scaled scores in each cognitive domain (episodic memory, processing speed, executive function, and working memory). Missing data points (<0–3% of scores on individual subtests) were imputed using the R program *missRanger* (Mayer and Mayer, 2019).

We then applied linear transformations to the unregressed omnibus and domain z-scores using multiple stepwise regression to create standardized residual scores using a conventional model with Age, Education, and Gender (AEG) as covariates. We also computed standardized residual scores using a comprehensive model that controlled for age, education, gender, age^2^, Vocabulary score, race (White, Asian, or African American), ethnicity (Hispanic or non-Hispanic), socioeconomic status, daily prescription medications, comorbidities, hours spent reading, hours spent using a computer, anxiety (GAD7), depression (GDS), and functional impairments (FS20). Stepwise linear regression was then used to isolate factors that contributed significantly (*p* < 0.01) to the model solutions after outliers (typically ∼3% of scores) were eliminated based on Cook’s Distance test (*D* > 4 SDs from the mean). Optimal solutions, based on Akaike Information Criteria (AIC), produced conventional models with 2–3 significant predictors and comprehensive models with 4–7 significant predictors. Both AEG and comprehensive models resulted in residuals with homogeneous variances, insignificant collinearity, and normal distributions.

As shown below, we have found that adding additional regressors (e.g., Vocabulary, race, computer use, reading, and GDS scores, etc.) significantly improves model fit compared to models using conventional covariates (e.g., AEG). Our research team is currently refining statistical models with conventional and comprehensive regressors to individual patient subtest scores to derive normative-based percentile and z-scores.

### CCAB performance–At-home vs. in the laboratory

All participants in the normative CCAB study were initially tested at-home, the majority during COVID research lockdowns. However, laboratory testing became possible for many participants at the 6-month retest, allowing the comparisons of in-lab and at-home results. We therefore compared the results of at-home and in-laboratory CCAB test administration in 276 participants who have completed retests at 6 months. A total of 57.1% tested at home and 42.9% in the laboratory. Omnibus z-scores were calculated using comprehensive regressors along with baseline performance measures from the initial three at-home test sessions. As shown in Table 4, mean (omnibus) z-scores, averaged over all measures, did not differ significantly as a function of test site [at-home = −0.04, in-lab = 0.05, t(274) = 0.85, NS], nor were significant differences seen in the mean domain z-scores for episodic memory, processing speed or executive function subtests. The equivalence of at-home and in-lab results likely reflects two CCAB design features: (1) CCAB verbal stimuli are presented at high intensities through noise-attenuating headphones that minimize the impact of environmental noise, and (2) The CCAB enables similar remote subject monitoring at both test sites.

**TABLE 4:** Mean z-scores for in-lab and at-home CCAB administration.

Location	Omni	Memory	Speed	Exec
In-lab	0.05	0.06	0.08	0.03
At-home	−0.04	−0.07	0.01	0.01

Data are from the 276 subjects who completed retests at 6-months. *t*-tests showed no significant inter-group differences for any measure.

### Preliminary results: the effects of COVID-19 infection on cognition in older adults

**FIGURE 5:**
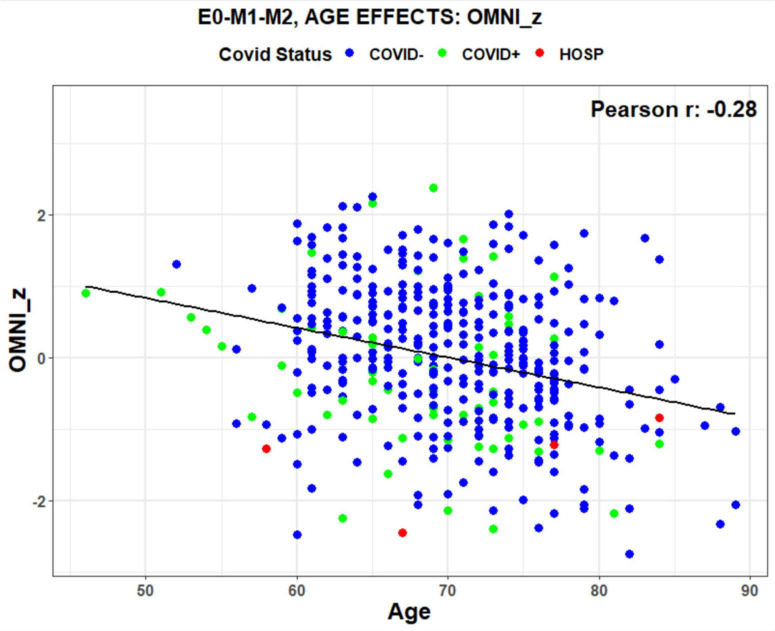
Unregressed omnibus z-scores as a function of age. Data points show COVID-19 status (blue = COVID-, green = COVID + , red = Hospitalized with COVID). The regression line shows a normal decline in performance with age. Data from 415 participants. E0-M1-M2 refers to the scores three test sessions that were included in the average.

We retrospectively identified participants in the normative population with and without a self-reported history of previous COVID infection. Table 4 shows the demographic characteristics of participants who were never infected (COVID-) vs. those who had recovered from previous COVID infections (COVID +). Unregressed omnibus z-scores, averaged over all subtests, were significantly lower in COVID + than COVID- participants [t(82) = −2.45, *p* < 0.02] as shown in Figure 5.

### Conventional vs. comprehensive regression models of COVID-19 effects

**FIGURE 6:**
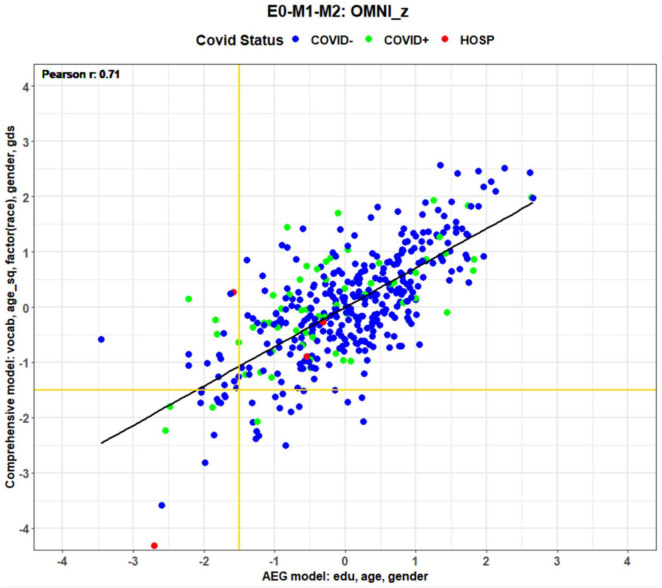
Omnibus z-scores from enrollment tests calculated with comprehensive and AEG regressors. Axis labels show significant predictors in order of significance (e.g., vocabulary was the most significant predictor in the comprehensive model). COVID status is coded by dot color.

Figure 6 shows the omnibus z-scores (averaged over all subtests) calculated with comprehensive and conventional (AEG) models. Predictors are shown on the axes in order of significance: Vocabulary, Age^2^, Race, Gender, GDS, hours reading, and daily prescription medications for the comprehensive model, and Education, Age, and Gender for the conventional AEG model. The comprehensive model accounted for more than twice as much variance (63%) as the AEG model (24%). Figure 6 shows a systematic discrepancy in model in z-scores for patients with COVID (green and red dots): their z-scores were higher when calculated with comprehensive than the AEG models, as reflected by the displacement above the regression line. The displacement reflects the contribution of significant demographic factors that were not included in the AEG model. For example, COVID + participants were disproportionately from minority communities and race was a significant predictor in the comprehensive model. Vocabulary scores, proxies for premorbid verbal intelligence, were the most significant covariate in the comprehensive model, and were also significantly lower (*p* < 0.01) in the COVID + group, as were weekly hours reading (*p* < 0.005), while GDS scores were higher (*p* < 0.05). Thus, correcting for pre-existing demographic differences (e.g., race, vocabulary, hours reading, and depression) disproportionately increased the z-scores of COVID + participants with the comprehensive model relative to those calculated with the conventional AEG model.

Table 5 shows the mean group z-scores of COVID- and COVID + participants analyzed with the AEG and comprehensive models. Group-mean z-scores calculated with the conventional AEG model were significantly reduced by COVID infection in all cognitive domains. In contrast, group-mean differences failed to reach statistical significance when analyzed with the comprehensive model. This reflects the fact that the comprehensive model reduced the contributions of pre-existing demographic factors that distinguished the groups.

**TABLE 5:** Mean z-scores for AEG and comprehensive (COMP) models.

Model	COVID	EM	PS	EF	WM	Omni
AEG	COVID−	0.04	0.06*	0.07**	0.05*	0.06*
	COVID+	-0.23	-0.31	-0.37	-0.29	-0.33
COMP	COVID−	0.01	0.02	0.03	0.02	0.01
	COVID+	-0.04	-0.09	-0.16	-0.09	-0.08

EM, episodic memory; PS, processing speed; EF, executive function; WM, working memory; Omni, omnibus. Significance of t-test comparisons of COVID- and COVID + groups:

*p < 0.05,

**p < 0.01. Data from 415 participants.

### COVID-19 severity effects

We next analyzed the correlations between domain scores and COVID severity among COVID + patients. COVID severity was scored using World Health Organization (WHO) classification: 0 = no infection, 1–3 = mild infection without hospitalization, 4–5 = hospitalization without assisted ventilation, 7–9 = hospitalization with assisted respiration, supplemented with adjustments for the severity of COVID symptoms in non-hospitalized patients. When analyzed with the conventional AEG model no correlations with COVID severity reached statistical significance. In contrast, when analyzed with the comprehensive model COVID severity correlated significantly with omnibus z-scores [*r* = −0.30, t(64) = −2.52, *p* < 0.02], episodic memory z-scores [*r* = −0.28, t(64) = −2.31, *p* < 0.05], and executive function z-scores [*r* = −0.27, t(64) = −2.24, *p* < 0.05]. Thus, these preliminary results suggest that the comprehensive model more accurately captured the specific effects of COVID infection (reflecting greater performance declines in patients with more severe infections), while the apparent COVID-related impairments seen in Table 5 with the conventional (AEG) model reflected the demographic differences between the COVID + and COVID- cohorts which obscured the relationship between cognitive performance and infection severity.

### Model effects on MCI classification

Different scoring models will also influence on the diagnosis of individual patients. For example, scores in the lower left corner of Figure 6 show participants with omnibus performance in MCI-performance range (i.e., global z-scores <−1.50). Horizontal and vertical gold lines show the MCI cutoffs (*z* = −1.50) for the comprehensive and AEG models, respectively. Fewer than one-third of MCI subjects were identically classified with the two models (i.e., below the horizontal gold line and to the left of the vertical gold line). Many participants showed MCI-level performance with the comprehensive model but not the AEG model (bottom center) and vice versa (top left). In some cases, score discrepancies were substantial. For example, one patient who had been hospitalized with COVID (red dot, left center of Figure 6) produced a z-score consistent with MCI (−1.58) when scored with the conventional AEG model, but a z-score of + 0.35 when scored with the comprehensive model.

In addition, we found that a higher percentage of non-white participants (14.8%) fell in the MCI range with the conventional AEG model than with the comprehensive model (9.3%). Correspondingly fewer white participants were classified in the MCI range with the AEG model (3.56%) than with the comprehensive model (5.93%). This finding is unsurprising because the comprehensive model factored out the influence of race and demographic factors correlated with race in the model solution. Hence, the comprehensive model not only produced a better fit to the data, but also reduced racial and ethnic biases. In summary, comprehensive regression models, made possible by the extensive CCAB questionnaire and rapid vocabulary subtest, appear to provide more precise and less biased estimates of cognitive performance than conventional models that account only for the influences of age, education, and gender on performance.

## Discussion

### A comparison of manual and computerized NPA

The CCAB, like other digital cognitive tests, builds upon the designs of manually administered paper-and-pencil tests but incorporates significant methodological improvements in test designs, response density, response quantification, stimulus delivery, and test scoring, while reducing examiner effects, racial bias, and improving test accessibility.

• **Test designs.** Computerized tests can incorporate adaptive staircase procedures, impossible with manual testing, that are more time-efficient and precise than non-adaptive tests (Bilder and Reise, 2019). CCAB subtests of vocabulary, choice reaction time (Woods et al., 2015a,g), spatial span (Woods et al., 2015b,d) and digit span (Woods et al., 2011a,b) use adaptive staircase procedures.
• **Increased response density.** CASR’s timestamped scoring of individual responses also makes it possible to reconfigure standard tests (e.g., Stroop, picture naming, symbol-number coding, etc.) to increase response density and evaluate temporal fluctuations in performance to improve the tests’ psychometric properties.
• **Response quantification.** In addition to core measures, digital tests can include supplementary measures that are difficult to obtain with manual tests. For example, CCAB verbal fluency scores include measures of response time as well as accuracy. Digital tests can also incorporate computational measures difficult to obtain with manual test administration. For example, the CCAB verbal fluency subtests includes scores of semantic organization and semantic clustering (Woods et al., 2016a) using Explicit Semantic Analysis (Gabrilovich and Markovitch, 2009). Similarly, logical memory scores include measures of speech rate (e.g., syllables per second) and computationally scored lexical features (e.g., word frequencies and type/token ratios). Comprehensive analyses are also possible for non-verbal tests. For example, CCAB Trail Making subtests include measures of movement time, drawing velocity, line directness, error-correction time, and the time required to connect individual line segments (Woods et al., 2015c).
• **Stimulus delivery.** In manual testing, inter-examiner and inter-session differences occur in speech rate, loudness, and articulatory intelligibility. Speech audibility will also be affected by the acoustics of the testing room and the degree of hearing loss, particularly in older participants and (Füllgrabe, 2020). Questionnaires showed that 41% of the CCAB normative population reported hearing loss, but only 27% reported using hearing aids. To compensate for hearing loss, the CCAB adjusts speech intensities based on speech recognition thresholds.
• **Test scoring.** Often, only Age and Gender are used in interpreting the results of manual NPAs. Thus, potentially important covariates including education, vocabulary, race, comorbidities, functional disabilities, intellectual activities (e.g., computer experience), and emotional state (e.g., depression) are not taken into consideration when interpreting scores. The comprehensive CCAB scoring model includes many pre-existing demographic factors that should be included to optimize the validity of test interpretation.
• **Examiner effects.** Examiner effects (e.g., average participants perform better with examiner A than examiner B) are evident in virtually all manually administered tests (Overton et al., 2016) and can be of substantial magnitude (Wiens et al., 1994). Less is known about Examiner x Participant interactions (e.g., examiner A elicits better performance from participant X than participant Y while the opposite is true for examiner B) which depend on the rapport of the examiner and the patient. Patient-examiner interactions may be particularly important in inter-racial testing situations, where previous studies have shown that conditions that maximize stereotype threat (white examiner, African American participant) can reduce African-American test scores by more than one standard deviation (Thames et al., 2013). The CCAB minimizes examiner effects by ethnoracial matching examiners and participants when possible and because subtests are largely self-administered with minimal video chat interactions during most tests.
• **Ethnoracial bias.** Few existing manual tests have robust norms for interpreting test results from racial and ethnic minorities. In addition, manual testing may disadvantage ethnoracial minority participants for several other reasons, including (1) Increased participant anxiety about testing in unfamiliar and alien surroundings: (2) Unacknowledged failures in understanding test instructions resulting in artifactually low scores; and (3) Examiner scoring bias, including unconscious bias due to differences in performance style and dialect. NBS is now testing minority populations to establish robust ethnoracial norms, including norms for Spanish-speaking Hispanics. The CCAB reduces ethnoracial bias because (1) CCAB tests can be administered in the familiar surroundings of the participant’s home rather than in an unfamiliar laboratory; (2) The CCAB assures that participants understand subtest instructions by requiring that participants perform training trials with sufficient accuracy. If gross failures occur early in a subtest, training trials and/or subtests can be readministered; (3) CCAB scoring is automated and objective, with CASR (and TRT review) reducing ethnoracial bias in verbal-test scoring, and (4) Ethnoracial examiner-patient compatibility can be optimized by flexible examiner assignment.
• **Test accessibility.** Digital tests can help to overcome the cost and efficiency barriers that limit access to manual NPAs. Manual testing resources are already inadequate to meet the growing NPA demands of the elderly population. The 5,700 practicing neuropsychologists in the US currently administer approximately 1M NPAs annually (Sweet et al., 2021). Each manually administered NPA requires an average of 10.4 h (Rabin et al., 2016) to obtain a patient history, select and administer tests, transcribe and score responses, interpret the results, write a report, and counsel the patient and family. As a result, manual NPA costs are high (mean $3500), only partially reimbursed by healthcare insurance, and often unavailable at Medicare and Medicaid reimbursement rates (Kurniadi and Davis, 2022). Moreover, manual testing resources are concentrated in major metropolitan areas, limiting access in exurban and rural communities (Sweet et al., 2021). Efficient computerized batteries, delivered remotely and scored automatically, can significantly increase access to underserved populations while reducing costs.

### “Gold standard” NPAs of the future

Improvements in software and technology now make it possible to create “cybertest” clones of existing “gold standard” manual cognitive tests that automate the administration and scoring. Cybertest clones should have superior psychometric properties compared to the original manual version because of the improved precision and reliability of test administration and the reduction of errors in response tallying and scoring. Moreover, clones can comprehensively analyze performance (e.g., clustering and switching in verbal fluency tests) and provide information about response latencies to individual stimuli that cannot be measured in manual tests. For example, the CCAB’s verbal learning subtest (the BAVLT) incorporates the design features of the California Verbal Learning Test (CVLT) (Delis et al., 1987) in a briefer test format. The BAVLT tallies scores automatically and displays realtime performance to the examiner during each trial, and automatically scores final results with exhaustive scoring metrics similar to those of the CVLT (e.g., semantic reorganization, primacy and recency effects, learning effects across trials, recognition testing, etc.). It also provides additional measures (e.g., response latencies and temporal clustering, etc.) that cannot be obtained with the CVLT. Moreover, because digital tests are largely automated they liberate the examiner to integrate the results of current and previous subtests during assessments, enabling the examiner to select and administer additional subtests when clinically indicated.

The demographic reality of an aging world will necessarily drive new assessment and diagnostic technologies that will change the face of neuropsychology and aging sciences. Dramatic increases in the older adult population will bring with it a sharp increase in age-related chronic diseases such as ADRD and cerebrovascular disease. Modern medicine’s ability to detect, diagnose, and treat these diseases will struggle to meet these growing assessment demands. Already, COVID forced many older adults to shelter-in-place and consequently adopt new social media technology to engage with their support networks, doctors, and services. These trends will continue in the future, as digital innovations (e.g., self-driving cars, digital care-giving assistants, etc.) increasingly impact the lives of older individuals. Digital NPAs will contribute to this trend, and will likely evolve rapidly with the integration of AI (Artificial Intelligence) models that can select and adjust subtest sequences in realtime, and assist in the interpretation of assessment results. Thus, future digital NPA batteries are likely to be increasingly adaptive and customized for each patient based on their health status, neuropsychiatric history, demographic characteristics, and realtime test performance.

The CCAB has been designed for use in research and clinical trials, with the long-term goal of providing clinical tests to patients throughout the US and overseas. NBS plans to license the CCAB to research users in 2024. Users will receive training and be provided with access to the deidentified and anonymized data in the CCAB’s normative database, including longitudinal test results. This will facilitate power estimations when designing new studies, enable small samples to be demographically matched with control subjects in the database, and provide a large additional normative sample against which targeted populations can be compared and the significance of hypothetical effect sizes evaluated.

Clinical trials will benefit from the increased sensitivity, comprehensiveness, and quality control of CCAB subtests compared to existing computerized or manual instruments, and by the fact that the CCAB can be efficiently administered at national and international trial sites by a core of experienced examiners. Moreover, the CCAB’s comprehensive scoring models, scalability, and high-test retest reliability provide unexcelled sensitivity to small treatment effects in longitudinal studies enabling significant reductions in require sample sizes.

California Cognitive Assessment Battery administration is flexible: It can be administered in patients’ homes or in the laboratory. Video/audio chat functions make it easy for the examiner to greet patients and check in with them as needed during the assessment. Many CCAB subtests adapt to a patient’s skill level, beginning with easy items and becoming more difficult if the patient is successful. Therefore, the patient is not made to struggle through difficult items that can make them feel frustrated and fatigued. Complete CCAB results can be viewed as soon as the test session is over, so that clinicians can integrate subtest results during test administration and discuss a patient’s strengths and weaknesses at the conclusion of the assessment. This contrasts to manual neuropsychological NPAs that take many hours to score and analyze and then weeks for another appointment to review the test results with patients and caregivers.

The CCAB has been in development over the last 10 years, and the current version has been extensively tested in more than 1,000 healthy participants from diverse backgrounds. Earlier versions of the CCAB were updated to include more representative stimuli (e.g., ethnically diverse faces in the Face-Name Binding subtest). In addition, NBS recently received NIH-NIA funding (R44AG080951) to support large scale normative data in historically excluded groups, including African Americans, Hispanics, and Asian Americans. Finally, the CCAB was recently translated into Spanish for the US Spanish-speaking population, and normative data collection with CCAB-Español is now underway.

## Limitations and future plans

### CCAB administration time

Customized assessments with selected CCAB subtests can be packaged in minutes, and new tests can be rapidly programmed for norming. In 2024, NBS plans to norm a 90-min version of the CCAB for single-session administration with the option of adding or removing subtests at the discretion of the examiner.

### Fatigue effects

Fatigue effects may degrade performance on CCAB subtests that occur later in the test sessions. The analysis of fatigue effects requires large normative samples of participants with different CCAB subtest orders. Ongoing research programs at UC Davis that use selected subtests presented in different order will provide preliminary insight into the magnitude of subtest-order effects.

### Limitations in patient observation

Because the CCAB examiner views the participant through the tablet’s camera the field of view is limited to the participant’s head and upper torso. The limited field of view complicates the detection of cheating (e.g., the participant’s hands are not always visible). Therefore, setup instructions specify that no paper or pencil be available in the test area. Clinically relevant observations of the participant’s mobility, neatness, and other characteristics are also limited by the field of view.

### At-home testing challenges

Although at-home and in-lab test administration produce similar average results (see above), in some assessments at-home distractions can occur (e.g., a doorbell, phone call, spouse) that jeopardize subtest validity. For example, if a disruption occurs in the middle of a subtest, the examiner can either abort the subtest or repeat the subtest when conditions permit. However, both solutions compromise data quality. Some unusual challenges have also occurred in the approximately 3000 at-home CCAB assessments that have been administered. For example, one participant fell asleep during a rest period (testing was resumed when he awakened), and, on another occasion “the cat got the mouse,” i.e., a pet cat damaged the cable connecting the mouse to the tablet (the mouse was replace).

### Cellular connection challenges

Most CCAB tests administered in participants’ homes have been administered using cellular connections, because older participants often find it challenging to retrieve their Wi-Fi passwords. Intermittent fluctuations in cellular connectivity have resulted in the temporary loss of video chat capability in approximately 10% of CCAB test sessions. In these cases, CCAB test administration (controlled by the tablet computer) is unaffected, and, in most cases, video chat capability can be restored. More problematically, cellular connection strength varies in different geographic locations. Indeed, at-home testing has proved impossible in several cases where cellular connection strength was inadequate, and participants lacked Wi-Fi access.

### Retest learning effects

Heathy participants generally show significant retest learning effects on repeated testing (Hassenstab et al., 2015; Soldan et al., 2017). Because reduced retest learning effects can identify older individuals with preclinical AD (Duff et al., 2018), retest effects have become a focus of recent AD clinical trials (Jutten et al., 2023). Large retest learning effects occur in many CCAB subtests and will be described in future publications.

### Geographically limited CCAB norms

California Cognitive Assessment Battery norms have been gathered from older participants residing in the San Francisco Bay Area. In the future, NBS plans to expand normative data collection nationwide to create more nationally representative norms that will also include younger participants.

### Lack of malingering subtests

While the CCAB does not currently include a specific malingering subtest, participants performing with suboptimal effort can be detected from the pattern of their responses on many CCAB subtests (Woods et al., 2011b, 2015a,b,c,d,e,g, 2016a,b, 2017; 2018; Hubel et al., 2013b).

## Conclusion

The CCAB is a comprehensive NPA battery that uses innovative technologies, test designs, and metrics to characterize cognitive functioning using at-home or in-laboratory administration. The CCAB runs on a tablet computer with built-in cellular connectivity to enable patient assessments of lower-income and un-housed individuals who often lack Wi-Fi access. The CCAB automates test administration and response tallying and incorporates comprehensive scoring models that more accurately predict the performance of individual patients that conventional models by incorporating additional demographic factors that significantly influence performance. CCAB norms from racially, ethnically, and linguistically diverse populations are now being gathered to permit valid test interpretation for patients from minority groups. Remote at-home, automated computerized, assessments of cognitive functioning are critically needed at the institutions like the Veteran Affairs (VA) healthcare system to enable clinical-grade NPAs to be administered in a patient-centered, efficient, and low-cost manner. In short, the CCAB holds the promise of providing access to state-of-the-art NPA assessment to underserved patients including those with mobility challenges and those living in rural and exurban areas.

## Data availability statement

The datasets presented in this article are not readily available because of legal and privacy issues: IRB approval at the VA did not allow data sharing. Requests to access the datasets should be directed to drdlwoods@neurobs.com.

## Ethics statement

The studies involving humans were approved by the VA Northern California Health Care System Institutional Review Board and by the Western IRB. The studies were conducted in accordance with the local legislation and institutional requirements. The participants provided their written informed consent to participate in this study. Written informed consent was obtained from the individual(s) for the publication of any identifiable images or data included in this article.

## Author contributions

DW: Conceptualization, Data curation, Formal analysis, Funding acquisition, Investigation, Methodology, Project administration, Resources, Software, Supervision, Validation, Visualization, Writing – original draft, Writing – review and editing. PP: Conceptualization, Data curation, Funding acquisition, Methodology, Project administration, Resources, Software, Supervision, Validation, Visualization, Writing – review and editing. DJ: Data curation, Formal analysis, Investigation, Project administration, Resources, Supervision, Writing – review and editing, TH: Conceptualization, Data curation, Formal analysis, Investigation, Methodology, Project administration, Software, Validation, Visualization, Writing – review and editing. KH: Data curation, Formal analysis, Investigation, Project administration, Supervision, Writing – review and editing. MB: Data curation, Formal analysis, Investigation, Project administration, Software, Supervision, Writing – review and editing. KG: Conceptualization, Data curation, Formal analysis, Project administration, Software, Supervision, Validation, Validation. GW: Investigation, Project administration, Resources, Software, Supervision, Writing – review and editing. JC: Data curation, Formal analysis, Investigation, Project administration, Supervision, Writing – review and editing. SL: Data curation, Formal analysis, Investigation, Project administration, Supervision, Validation, Writing – review and editing. BC: Data curation, Investigation, Project administration, Resources, Supervision, Writing – review and editing. KS: Data curation, Formal analysis, Investigation, Supervision, Writing – review and editing. MS: Data curation, Project administration, Writing – review and editing. JB: Conceptualization, Data curation, Formal analysis, Funding acquisition, Investigation, Methodology, Project administration, Resources, Supervision, Validation, Writing – original draft, Writing – review and editing.

## Footnotes

1
freerice.com
